# Migraine as a Risk Factor for Peripheral Artery Occlusive Disease: A Population-Based Cohort Study

**DOI:** 10.3390/ijerph17228549

**Published:** 2020-11-18

**Authors:** Fu-Hsuan Kuo, Chia-Yi Lee, Ju-Pi Li, Jui-Fu Chung, Yu-Hsun Wang, Ming-Ju Hsieh, Shun-Fa Yang

**Affiliations:** 1 Institute of Medicine, Chung Shan Medical University, Taichung 402, Taiwan; kfs0611@gmail.com; 2 Neurological Institute, Taichung Veteran General Hospital, Taichung 407, Taiwan; 3 Department of Ophthalmology, Show Chwan Memorial Hospital, Changhua 500, Taiwan; ao6u.3msn@hotmail.com; 4 School of Medicine, Chung Shan Medical University, Taichung 402, Taiwan; d888203@gmail.com; 5 Department of Pediatrics, Chung Shan Medical University Hospital, Taichung 402, Taiwan; 6 Radiology Division, Chiayi Branch, Taichung Veteran General Hospital, Chiayi 600, Taiwan; jeff110175@gmail.com; 7 Department of Medical Research, Chung Shan Medical University Hospital, Taichung 402, Taiwan; cshe731@csh.org.tw; 8 Cancer Research Center, Changhua Christian Hospital, Changhua 500, Taiwan; 9 Graduate Institute of Biomedical Sciences, China Medical University, Taichung 404, Taiwan

**Keywords:** migraine, peripheral occlusive artery disease, atherosclerosis, cardiovascular disease

## Abstract

We aim to evaluate the development of peripheral occlusive artery disease (PAOD) in patients with migraine by using the National Health Insurance Research Database in Taiwan. A retrospective cohort study was conducted and individuals with diagnostic codes of migraine were enrolled in the study group after excluding those diagnosed with PAOD before the index date. Each subject with migraine was propensity-score matched to another non-migraine patient and the latter served as the control group. A total of 37,288 patients were finally enrolled in the groups. The primary outcome was set as the development of PAOD between the two groups while multiple possible risk factors, including demographic data and comorbidities, were analyzed via the Cox proportional hazards regression. There were 885 and 530 PAOD events in the study and control groups, and the study group had a significantly higher adjusted hazard ratio (1.65, 95% confidential interval: 1.48–1.84, *p* < 0.001), and the cumulative incidence also revealed a correlation between migraine and PAOD. Other potential risk factors related to the existence of PAOD include age, hypertension, chronic kidney disease, diabetes mellitus, coronary artery disease, stroke, and asthma. For individuals without certain systemic diseases including hypertension, chronic liver disease, chronic kidney disease, coronary artery disease, stroke, asthma, or heart failure, the hazard ratio of subsequent PAOD was significantly higher in the migraine patients than that in the non-migraine individuals (all *p* < 0.001). In conclusion, the presence of migraine is a significant risk factor for the development of subsequent PAOD.

## 1. Introduction

Migraine is one of the most common neurological disorders in the world [1], with a global prevalence about 14.4% which the pravalence in the female population is nearly 2-fold than that in the male populaiton [2]. The migraine is characterized by unilateral headache, pulsating quality, moderate or severe intensity, and associated with symptoms such as photophobia, phonophobia, nausea, and different types of aura [3]. Several studies have pointed out that the migraine may cause a huge socioeconomic burden in the world. According to the global burden of disease research in 2015, the migraine was one of the chronic disease that affected more than 10% of people worldwide [4], and is seventh of the leading 30 causes of global years lived with disability [4]. In addition, approximately one-third of migraineurs said that they worried about long term financial security of themselves or their family owing to their headaches [5]. For the objective index of migraine influence such as the payment, the annual financial cost of migraine is estimated to exceed US$20 billion [6]. 

Peripheral artery occlusive disease (PAOD), also named as peripheral arterial disease, peripheral vascular disease, and arteriosclerosis obliterans, is most commly due to atherosclerosis, which may causes stenosis or occlusive of artery and presented as intermittent claudication and critical limb ischemia [7]. Regarding the predisposing factors for PAOD, age contributes to the development of PAOD significantly, as a majority of PAOD cases occurred in the patiets’ 60s and 70s [7,8]. In addition, the male gender experiences more severe or symptomatic PAOD condition compared to the female population [8]. The other known risk factors for PAOD include cigarette smoking, diabetes mellitus, hypertension, hyperlipidemia, coronary artery disease, and cerebrovascular disease such as stroke [8,9,10,11,12,13].

Although it remains contraverisal [14], there are increasing evidences that migraine correlates to the development of cerebrovascular disease [15,16]. In a population-based cohort study, patients with migraine had higher prevalence of cerebellar infarction than controls with a higher adjusted odds ratio [15]. Another meta-analysis demonstrated that the pooled adjusted odds ratio of ischemic stroke was more than 2-fold higher in patients with migraine than the non-migraine patients [16]. In the last 5 years, cumulative cohort studies revealed that migraine may increase the risk of ischemic stroke [17,18,19,20], and the migraine is still a significant risk factor for stroke even after adjusting the effect of multiple covariates including menopausal status, postmenopausal hormone use, oral contraceptive use, and aspirin use [17]. Although the association between migraine and ischemic stroke has been reported, research on migraine and PAOD is still limited with only few studies indicating such possibility without surveying the time sequence between migraine and PAOD [21,22]. Since both the migraine and PAOD share similar feature of vascular abnormality [9,23], and ischemic stroke is correlated to the occurrence of not only migraine but also PAOD [12,16], the migraine may be a indicator of subsequent PAOD which still needs investigation.

Consequently, we aim to survey the correlation of migraine to the development of subsequent PAOD by using the National Health Insurance Research Database (NHIRD) in Taiwan. Moreover, other potential risk factors of PAOD were also analyzed in the multivariable model. To our knowledge, the current study is a preliminary research that attempt to evaluate whether the migraine can be served as an early sign of PAOD.

## 2. Materials and Methods 

### 2.1. Ethics Declaration and Data Resource

This retropective population-based cohort study was adhered to the declaration of Helsinki in 1864 and its late amedment, and the current study was also approved by the National Health Insurance Administration and the Institutional Review Board of Chung Shan Medical University Hospital (Registration Number: CSMUH CS17089). The need for informed consent was waived by the above two institutions. We analyzed data from the data set of the NHIRD, which was released by the Taiwan National Health Research Institutes. The NHIRD is an electronic database developed according to the National Health Program, which contains more than 99% of Taiwan’s population. In the current study, we obtained data from the Longitudinal Health Insurance Database 2005 version, which included data on two million patients randomly sampled from the NHIRD institution from the year 2005 and linked from 1 January 2000 to 31 December 2015. Both the International Classification of Diseases, Ninth Revision and Tenth Revision were used for disease diagnosis in the current study.

### 2.2. Patient Selection

Patients were defined as having newly-diagnosed migraine if they had received migraine-related diagnostic codes in more than three outpatient visits or one admission according to their medical records. The index date was set as the date on which the first diagnosis of migraine was received. To accurately elucidate the association between migraine and PAOD, patients with diagnosis of PAOD before the index date were excluded. For the comparison, a patient with migraine was matched to 10 patients without migraine via age and gender initially. The latter constituted the control group, then each patient in the study group was propensity score-matched with another non-migraine patient in the control group considering the demographic data and co-morbidities (shown in the following section). For the subgroup analysis, subjects in the study group were divided into different subgroup via age (20–40, 40–65, and those older than 65 years old), gender, and systemic co-morbidities. All these subgroups in the study group were compared to the patients in the control group with same character regarding the development of PAOD in the analysis.

### 2.3. Main Outcome Measurement

The development of PAOD which according to the diagnostic codes after index date was defined as the primary outcome for the current study. About the diagnostic criteria of PAOD, components including the presence of at least one PAOD-related symptom like the claudication (painful cramping) after physicial activity that invlove the lower limbs, lower limbs numbness, lower limbs coldness, and lower limbs weakness. In addition, the following signs are also used to diagnosed PAOD: Hypotension of the lower limbs, change of color of lower limbs, hair loss of lower limbs, presence of sores or poor wound healing at the feet, the presence of bruits, and a weak pulse in the lower extremities. In a patients with both PAOD-related symptoms and signs, the diagnosis of PAOD would be made if the Ankle-Brachial Index was lower than 0.9. For accuracy of the diagnosis, subjects were included only if they had at least three outpatient visits or more than one admission due to PAOD in their medical records.

### 2.4. Demographic Variables and Co-Morbidities

To standardize the health status of all the participants in this study, the following systemic co-morbidities were included in both the matching process and the multivariable analysis: Hypertension, hyperlipidemia, chronic liver disease, chronic kidney disease, diabetes mellitus (DM), chronic obstructive pulmonary disease (COPD), coronary artery disease, stroke, asthma, and heart failure according to the related diagnostic codes. We longitudinally traced the patient’s medical profile from the index date to the date of PAOD diagnosis, the date of death, or until 31 December, 2015, which meant the end of the NHIRD.

### 2.5. Statistical Analysis

The SAS 9.4 version (SAS Institute Inc, Cary, NC, USA) was applied for all the statistical analyses used in the current study. At first, the migraine patients were matched with a 1:10 ratio by age and gender to non-migraine subjects, then a 1:1 ratio by propensity score was performed again between the two populations to yield the study and control groups. To compare the characteristics of migraine and non-migraine groups, Chi-square test for categorical variables, and Student’s *t*-test for continuous variables which fitted normal distribution were used. Then the Cox proportional hazards regression was used to compute the adjusted hazard ratios (HR) of migraine and other potential risk factors for PAOD occurrence via incorporating those aforementioned demographic data as well as prominent systemic comorbidities in the analysis model. Besides, the Kaplan–Meier curves were drawn to demonstrate the cumulative probability of PAOD development between the study group and the control group, and the log-rank test was applied to determine whether there was a significant difference between the two groups. In the subgroup analysis, the IDs between the patients in study and control groups with different clinical characters were also evaluated by the multivariable analysis. The ethnicity was not considered a covariate in the current study because most of subjects (more than 98%) in Taiwan are Han Taiwanese. Statistical significance was set at *p* value lesser than 0.05. 

## 3. Results

**Figure 1:**
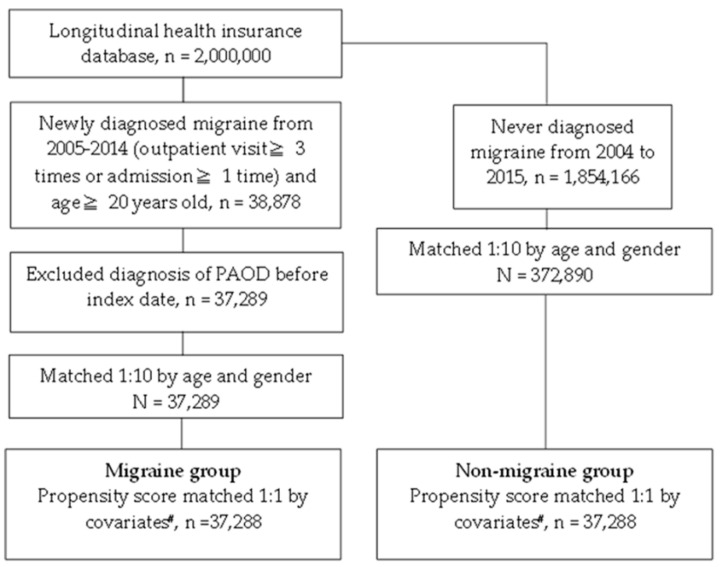
The flowchart of patient selection. *n*: Number; PAOD: Peripheral artery occlusive disease; # include age, gender, hypertension, hyperlipidemia, chronic liver disease, chronic kidney disease, diabetes, chronic obstructive pulmonary disease, coronary artery disease, stroke, asthma, and heart failure.

After the selection, a total of 37,288 patients were included in the study group, while another 37,288 subjects were enrolled in the control group. The flowchart of subject selection is shown in Figure 1. Besides, there is no difference in track time and time to event between two groups: The mean track time is 6.2 ± 2.9 years in both migrain and non-migraine group (*p* = 0.981) while the time to event in migraine group and non-migraine group are 3.4 ± 2.6 years versus 3.6 ± 2.6 years, respectively (*p* = 0.228). Due to the propensity score-matching process, there were no difference concerning demographic data as well as systemic co-morbidities between the study and control groups (Table 1).

**Figure 2:**
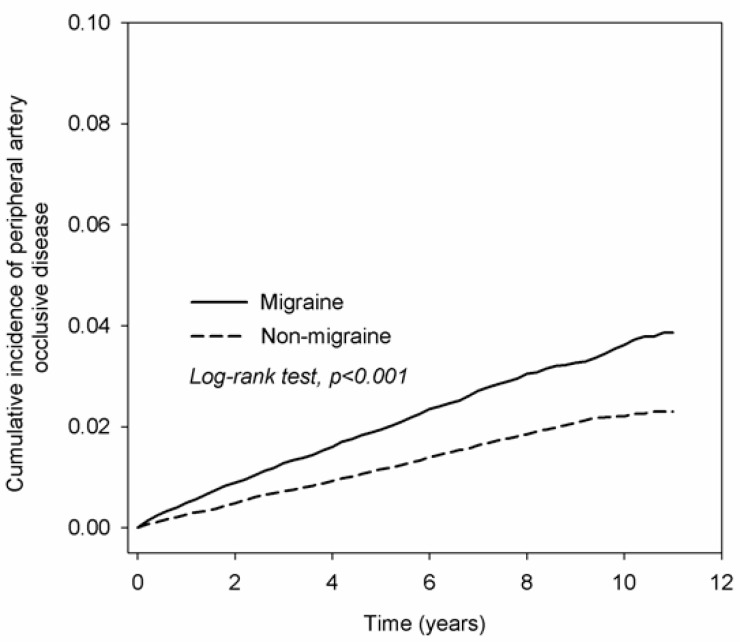
The Kaplan–Meier curves with a cumulative incidence of peripheral artery occlusive disease between the study and control groups.

After the whole follow-up period, there were 885 and 530 PAOD events in the study group and control group, respectively. It shows that the ID of PAOD significantly increased in the study group than that in the control group (adjusted HR: 1.65, 95% CI: 1.48–1.84, *p* < 0.001) after adjusting multiple covariates. Moreover, the ID of PAOD also increased in patients with older age compared to patients aged younger than 40 years old (*p* < 0.001), hypertension (*p* < 0.001), chronic kidney disease (*p* < 0.001), DM (*p* < 0.001), coronary artery disease (*p* < 0.001), stroke (*p* = 0.005), and asthma (*p* = 0.026) (Table 2). On the other hand, a higher cumulative incidence of PAOD in the study group was also found compaired with non-migraine subjects according to the Kaplan–Meier curves (log-rank *p* < 0.001, Figure 2).

In the subgroup analysis, the HR of PAOD is higher in the migraine individuals than that in the non-migraine population in all age subgroups, especially in the subgroup aged 20–40 years old (*p* for interaction = 0.001). The risk of PAOD were higher in both male and female with migraine (both *p* < 0.001) compared to non-migraine patients. About the analyses for systemic co-morbidities, the patients with migraine showed a higher incidence of PAOD compared to non-migraine individuals whether the following diseases were presented or not (all *p* < 0.05): Hypertension, hyperlipidemia, DM, and COPD. In addition, the risk of PAOD was significantly higher in individuals with migraine than those without migraine if that population is without any of the following co-morbidities (all *p* < 0.001): Chronic liver disease, chronic kidney disease, coronary artery disease, stroke, asthma, or heart failure. The other details of subgroup analyses are listed in Table 3.

## 4. Discussion

In the current study, we found the increasing risk of PAOD in the presence of migraine diagnosis after adjusting for multiple potential risk factors. The other risk factors which elevate the risk of PAOD include age, hypertension, chronic kidney disease, DM, coronary artery disease, stroke, and asthma.

Whether migraine is a risk of PAOD is conflicting in the previous studies. In a previous study, patients with migraine had decreased values of Ankle-Brachial Index compared to patients without migraine [21]. Nevertheless, this study was small in sample size with only 50 patients with migraine in the study group and 38 subjects in the control group [21]. In addition, the risk of PAOD was increased in the migraine group compared to the non-migraine patients with an odds ratio of 2.69 in a case-control study [22]. However, some preceding studies showed that migraine had no significant association with PAOD in overall analysis, and only those individuals with aura-related migraine were at increased risk of PAOD development [19,24]. Also, a Japanese case-control study showed that there was no difference of Ankle-Brachial Index between the migraine and non-migraine group [25]. In the current study, we demonstrated that migraine may have made an important contribution to the development of PAOD above and beyond traditional risk factors (*p* < 0.001). Moreover, we only considered the PAOD that occurred after the migraine episodes and could partially illustrate the time sequence between migraine and subsequent PAOD, which had seldom been reported in previous studies. Migraine has long-term been considered as a primary headache rather than a disease with cardiovascular comorbidity [1,2]. Still, according to the results of the current study, it is worth emphasising that migraine may link with risk of PAOD, even among patients without DM or hypertension.

The exact mechanism leading to the correlations between migraine and PAOD needs further investigation. In general, atherosclerosis has always been a main concept of PAOD [9,26,27], and PAOD was used to be thought as a comorbidity of stroke and coronary artery disease [8]. In a review article, endothelial dysfunction predisposed to atherosclerosis have been thought to be an important role between migraine and peripheral vascular disease, but controversial findings were found in several studies [28]. However, in the current subgroup analysis, we found that patients with migraine were associated with higher HR of PAOD even in patients aged 20–40, in groups without hypertension, DM, hyperlipidemia, coronary artery disease, and stroke (all *p* < 0.001), while all the above subgroup is not a high-risk group for PAOD. Moreover, the risk of PAOD in females with migraine was not lower than that in the males with migraine while being male is a risk factor to PAOD [8]. These findings may result from the following speculation: Migraine itself can be correlated and shares some similar mechanisms to subclinical atherosclerosis according to previous experience [29]. In addition, the vascular endothelial dysregulation was observed in both the migraine and PAOD [25,30]. Thus, the presence of migraine indicates a more vulnerable vascular system and PAOD has a higher chance of developing even in low-risk populations like people of a younger age and female gender. The explanation for the non-significant influences of migraine in subgroup with many systemic diseases is that the patient number in the subgroup with systemic co-morbidities were small, which may contribute to the statistical bias. In either condition, these concepts need further reasearch to validate.

Based on the multivariate analysis, other potential risk factors related to the existence of PAOD include age, hypertension, chronic kidney disease, DM, coronary artery disease, stroke, and asthma, which is grossly similar to the pervious experience except chronic kidney disease and asthma [7,8,9,10]. A meta-analysis studied the risk of PAOD in patients with chronic kidney disease revaled that they had higher HR of PAOD according to the estimated glomerular filtration rate [31]. Although we did not analysis the stage of chronic kidney disease, the overall adjusted HR for PAOD in subjects with chronic kidney disease was significantly higher in the current study. Besides, Yao et al. found patients with asthma are at higher risk of PAOD compaired with those without asthma [32], which is conflicting to the findings of the current study. The mechanism for the correlation between PAOD and asthma is uncertain, but inflammation is a crucial pathway in asthma which also provides a possible role as the pathogenesis of atherosclerosis [33,34,35].

Concerning the degree of generalization of the findings in the current study, more than 95 perent of inhabitants in Taiwan belong to the Han ethnicity which is also the major ethnicity in China, Hong Kong, Macau, and Singapore, and accounts for approximately one-fifth of the global human population [36]. In addition, Taiwan, China and the countries in Southeast Asian have a human population of 2 billion and the diet and cooking styles among these countries are similar [37]. Consequently, the generalization of the current study could be adequate since the characteristics of the Taiwanese are similar to a medium-to-large population in other regions of the world.

There are several limitations in the current study. The observational and retrospective design may restrict the accuracy and precision of the results. Furthermore, we applied claimed data rather than real medical records thus we may missing certain important messages including the classification of migraine, the severity of migraine, and the treatment of migraine. Besides, the influence of migraine on PAOD may be overestimated because we did not include cigarette smoking as a covariate because physician in Taiwan rarely entered smoking history in the health insurance system due to no related claimed codes. As a consequence, whether a patient is cigarette consumpter can only be investigated via viewing the real medical record. Nevertheless, the content for medical records is not available in the National Health Insurance Research Database thus including cigarette smoking into the multivariable analysis could not be done in the current study. On the other hand, the effect of triptans application, a medication that is used to treat migraine and could relate to the cerebrovascular disease, was not considered in the multivariable analysis thus the effect of migraine on PAOD development might be overestimated. Still, the formal approvals for the two triptans in Taiwan, including the Sumatriptan and Rizatriptan, were effective from 2015, which is a very late time period in the National Health Insurance Research Database which started from 2000 and ended in 2016. As a consequence, the enrollment of triptans in the analysis may not influence the whole study results to a large degree.

## 5. Conclusions

In conclusion, migraine increases the risk of PAOD after adjusting for multiple potential risk factors and the incidence of PAOD is positively correlated to the disease interval of migraine. Furthermore, the significant time sequence between migraine and subsequent PAOD was observed which imply the migraine may serves as a early predictor for the development of PAOD. Since the PAOD can lead to severe limb ischemia and permenant disability, subjects with prolonged migraine should be referred to cardiovascular department periodically to monitor the possible development of PAOD and manage it in the early phase. Further prospective studies to evaluate whether the treatment of migraine will influence the risk of subsequent PAOD development is advocated. 

## Figures and Tables

**Table 1:** Basic characteristics of migraine and non-migraine.

Characters	Migraine (*n* = 37,288)		Non-Migraine (*n* = 37,288)		*p* Value
	*n*	%	*n*	%	
Age					0.981
20–40	15,509	41.60	15,490	41.50	
40–65	17,947	48.10	17,952	48.10	
≥65	3832	10.30	3846	10.30	
Gender					0.894
Female	27,619	74.10	27,635	74.10	
Male	9669	25.90	9653	25.90	
Hypertension	5532	14.80	5536	14.80	0.967
Hyperlipidemia	2562	6.90	2560	6.90	0.977
Chronic liver disease	1281	3.40	1278	3.40	0.952
Chronic kidney disease	193	0.50	180	0.50	0.500
DM	1882	5.05	1884	5.05	0.973
COPD	712	1.90	720	1.90	0.831
Coronary artery disease	1398	3.70	1386	3.70	0.817
Stroke	1115	3.00	1121	3.00	0.897
Asthma	751	2.00	745	2.00	0.875
Heart failure	212	0.60	202	0.50	0.622

DM: Diabetes mellitus; COPD: Chronic obstructive pulmonary disease.

**Table 2:** Risk of peripheral artery occlusive disease between migraine and non-migraine group in Cox proportional hazard model.

Parameters	No. of Migraine	No. of PAOD	Observed Person-Years	ID	Crude HR	95% CI	*p* Value	Adjusted HR ^†^	95% CI	*p* Value
Migraine										
No		530	229,663	2.3	1			1		
Yes		885	229,644	3.9	1.67	1.50–1.86	<0.001 *	1.65	1.48–1.84	<0.001 *
Age										
20–40	15,509	161	194,100	0.8	1			1		
40–65	17,947	792	222,342	3.6	4.29	3.62–5.09	<0.001 *	3.65	3.08–4.34	<0.001 *
≥65	3832	462	42,865	10.8	12.92	10.80–15.45	<0.001 *	7.93	6.51–9.66	<0.001 *
Gender										
Female	27,619	982	343,677	2.9	1			1		
Male	9669	433	115,630	3.7	1.31	1.17–1.46	<0.001 *	1.11	0.99–1.25	0.064
Hypertension	5532	506	63,384	8.0	3.46	3.10–3.86	<0.001 *	1.37	1.20–1.56	<0.001 *
Hyperlipidemia	2562	210	28,316	7.4	2.63	2.27–3.05	<0.001 *	1.10	0.94–1.30	0.222
Chronic liver disease	1281	89	15,173	5.9	1.96	1.58–2.43	<0.001 *	1.24	1.00–1.54	0.053
Chronic kidney disease	193	36	1563	23.0	7.47	5.36–10.41	<0.001 *	2.68	1.92–3.76	<0.001 *
DM	1882	224	20,416	11.0	4.01	3.48–4.63	<0.001 *	1.70	1.45–1.99	<0.001 *
COPD	712	68	8428	8.1	2.70	2.11–3.44	<0.001 *	1.15	0.89–1.48	0.288
Coronary artery disease	1398	183	16,216	11.3	4.05	3.46–4.73	<0.001 *	1.48	1.25–1.75	<0.001 *
Stroke	1115	119	12,257	9.7	3.33	2.76–4.01	<0.001 *	1.33	1.09–1.61	0.005 *
Asthma	751	61	8980	6.8	2.26	1.75–2.92	<0.001 *	1.35	1.04–1.76	0.026 *
Heart failure	212	31	2022	15.3	5.00	3.50–7.13	<0.001 *	1.44	1.00–2.08	0.0502

PAOD: Peripheral artery occlusive disease; DM: Diabetes mellitus; COPD: Chronic obstructive pulmonary disease; ID: Incidence density (per 1000 person-years); CI: Confidence interval; HR: hazard ratio; † adjusted for migraine, age, gender, hypertension, hyperlipidemia, chronic liver disease, chronic kidney disease, diabetes, COPD, coronary artery disease, stroke, asthma, and heart failure.; * denotes significant difference between the two groups.

**Table 3:** Subgroup analysis of risk of peripheral artery occlusive disease between migraine and non-migraine group.

Parameters	Migraine		Non-Migraine		HR	95% CI	*p* Value
	*n*	No. of PAOD	*n*	No. of PAOD			
**Age**							
20–40	15,509	122	15,490	39	3.13	2.18–4.49	<0.001 *
40–65	17,947	491	17,952	301	1.63	1.41–1.88	<0.001 *
≥65	3832	272	3846	190	1.42	1.18–1.71	<0.001 *
*p* for interaction = 0.001 *							
Gender							
Female	27,619	608	27,635	374	1.63	1.43–1.85	<0.001 *
Male	9669	277	9653	156	1.76	1.45–2.15	<0.001 *
*p* for interaction = 0.507							
Hypertension							
No	31,756	590	31,752	319	1.86	1.62–2.13	<0.001 *
Yes	5532	295	5536	211	1.37	1.15–1.64	<0.001 *
*p* for interaction = 0.008 *							
Hyperlipidemia							
No	34,726	757	34,728	448	1.69	1.5–1.9	<0.001 *
Yes	2562	128	2560	82	1.55	1.17–2.04	0.002 *
*p* for interaction = 0.551							
Chronic liver disease							
No	36,007	835	36,010	491	1.70	1.52–1.9	<0.001 *
Yes	1281	50	1278	39	1.21	0.8–1.84	0.373
*p* for interaction = 0.121							
Chronic kidney disease							
No	37,095	869	37,108	510	1.71	1.53–1.9	<0.001 *
Yes	193	16	180	20	0.65	0.34–1.26	0.207
*p* for interaction = 0.004 *							
DM							
No	35,406	754	35,404	437	1.73	1.53–1.94	<0.001 *
Yes	1882	131	1884	93	1.39	1.07–1.82	0.015 *
*p* for interaction = 0.147							
COPD							
No	36,576	841	36,568	506	1.66	1.49–1.86	<0.001 *
Yes	712	44	720	24	1.86	1.13–3.06	0.014 *
*p* for interaction = 0.653							
Coronary artery disease							
No	35,890	784	35,902	448	1.75	1.56–1.97	<0.001 *
Yes	1398	101	1386	82	1.22	0.91–1.63	0.190
*p* for interaction = 0.022 *							
Stroke							
No	36,173	813	36,167	483	1.69	1.51–1.89	<0.001 *
Yes	1115	72	1121	47	1.44	0.997–2.08	0.052
*p* for interaction = 0.400							
Asthma							
No	36,537	847	36,543	507	1.67	1.5–1.87	<0.001 *
Yes	751	38	745	23	1.59	0.95–2.66	0.081
*p* for interaction = 0.865							
Heart failure							
No	37,076	871	37,086	513	1.70	1.52–1.89	<0.001 *
Yes	212	14	202	17	0.72	0.36–1.47	0.368
*p* for interaction = 0.016 *							

PAOD: Peripheral artery occlusive disease; DM: Diabetes mellitus; COPD: Chronic obstructive pulmonary disease; CI: Confidence interval; HR: Hazard ratio; * denotes significant difference between the two groups.
